# Fat, sex and caspase-2

**DOI:** 10.1038/cddis.2016.40

**Published:** 2016-03-03

**Authors:** C H Wilson, L Dorstyn, S Kumar

**Affiliations:** 1 Centre for Cancer Biology, University of South Australia, Adelaide, SA 5001, Australia

Caspases are a family of cysteine-dependent aspartate-specific proteolytic enzymes, known best for their roles in cell death and immune responses.^1^ Caspase-2, one of the first members of this family to be identified, is the most evolutionary conserved caspase, sharing strong homology with CED-3 in *Caenorhabditis elegans* and Dronc in *Drosophila.*^2^ As compared with other initiator caspases, caspase-2 is activated by dimerization and autocatalytic cleavage events.^1^ It contains a caspase-activation and recruitment domain (CARD) that facilitates its dimerization and activation.^1^ Comprising a nuclear localization signal in its prodomain region, it is the only caspase to be found localized in both cytoplasm and nucleus.^1^ Despite being a protease identified >20 years ago, knowledge of the *in vivo* caspase-2 substrates is limited, and thus the precise mechanism of its function remains to be fully understood.

Although caspase-2 is activated in response to a number of apoptotic stimuli, particularly those pertaining to stress (oxidative, genotoxic and metabolic),^1, 3, 4^ the evidence for an essential role in apoptosis *in vivo* is still limited, given that the caspase-2-deficient mice (*Casp2*^*−/−*^) are developmentally normal with no overt apoptotic defects. Through studies using *Casp2*^*−/−*^ mice, our group and others have demonstrated important roles for caspase-2 in apparently diverse functions, such as tumor suppression, regulation of oxidative stress response pathways and aging.^5, 6, 7^ In the absence of external stimuli, *Casp2*^*−/−*^ mice display a mild phenotype, of early onset-aging, decreased maximal body mass and bone density, and altered body composition (decreased fat mass).^6^ In male mice (aged 18–24 months), but not females, we observed a decrease in epidermal muscle mass;^5^ however, no one had described gender-specific differences in caspase-2 function until we published our recent study in *Cell Death Discovery.*^8^

In a previous study, utilizing proteomic and metabolomic analysis of liver and serum from young (6–9 weeks) and aged male *Casp2*^*−/−*^ mice, we identified a number of altered metabolites and pathways indicative of altered lipid metabolism and glucose homeostasis.^9^ These included a decrease in free fatty acids (FFAs), glycerol-3 phosphate, NADPH, altered mitochondria function and decreased blood glucose in the fed and fasted states (Figure 1).^9^ In addition, aged *Casp2*^*−/−*^ mice showed resistance to the development of age-induced glucose intolerance. Evidence supporting links to metabolism have also been provided by some other studies including regulation of human *CASP2* by sterol-regulatory element-binding protein,^10^ activation of caspase-2 in response to metabolic stress^1^ and, more recently, involvement of caspase-2 in lipoapoptosis.^11^

In our study,^8^ we further investigated the *in vivo* role of caspase-2 in metabolism by making the aged male and female *Casp2*^*−/−*^ mice fast for 18 h. Although gender-specific differences in metabolism are well established, our study is the first to demonstrate involvement of the caspase family in these differences. We demonstrate that the improved glucose homeostasis in aged male *Casp2*^*−/−*^ mice appears to be independent of insulin, and show that blood glucose levels are not altered in female *Casp2*^*−/−*^ mice (Figure 1).

Fasting is a form of nutritional stress resulting in glycogen depletion, breakdown of stored fat depots (lipolysis), autophagy and protein degradation as a means of providing energy for survival. We show that caspase-2 affects the response to fasting in a sex-specific manner, differentially altering loss of body mass, adipocyte cell size and the underlying molecular pathways. In male mice, loss of *Casp2* enhanced the fasting-induced decrease in liver mass and enhanced lipolysis as indicated by decreased adipocyte size and increased serum FFA (Figure 1). In female mice, loss of *Casp2* resulted in more significant loss in total body weight but not liver mass, and, interestingly, a decrease in adipocyte size was observed in both the fed and fasted states relative to WT. Gene expression analysis of white adipose tissue revealed potential differences in the utilization of FA between male and female *Casp2*^*−/−*^ mice. Loss of *Casp2* enhanced fasting-induced autophagy in both male and female mice (Figure 1). The lack of a gender difference in autophagy enhancement suggested that autophagy was a not a main reason for the altered glucose homeostasis in male *Casp2*^*−/−*^ mice, and further studies are required to investigate the causes of this.

Despite this study that clearly demonstrates an *in vivo* involvement of caspase-2 in the metabolism of lipids and suggests sex-specific regulation of glucose homeostasis and lipid metabolism, the mechanism of caspase-2 function remains unknown. Of the limited number of identified caspase-2 substrates, none appear to be associated with or explain its metabolic function. Further work will also be required to determine whether the function of caspase-2 in the regulation of lipolysis involves its catalytic activity (i.e., substrate/single protein cleavage). An interesting concept is that the role of caspase-2 in regulating metabolism may influence its tumor suppressor function.^12, 13^ However, the fact that whether the putative functions of caspase-2 in metabolism and tumor suppression are linked awaits future studies.

## Figures and Tables

**Figure 1:**
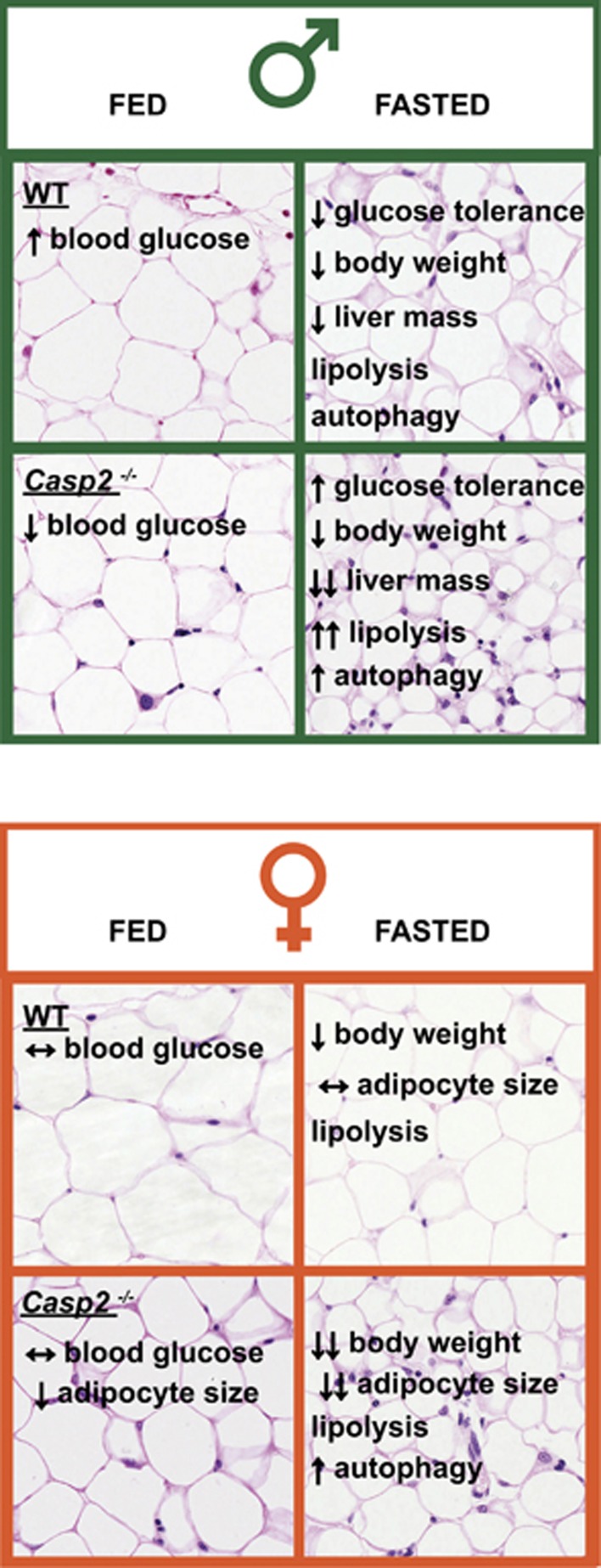
Sex-specific differences in caspase-2-deficient mice. The figure summarizes key metabolic differences between male and female WT and *Casp2*^*−/−*^ mice in the fed and fasted states. Upper panel (green) shows the key differences in male mice. Lower panel (orange) shows key differences in female mice. Background of each quadrant displays representative histological image of white adipose tissue stained with H&E to demonstrate differences in adipocyte size
